# Upper limb muscle atrophy associated with in-hospital mortality and physical function impairments in mechanically ventilated critically ill adults: a two-center prospective observational study

**DOI:** 10.1186/s40560-020-00507-7

**Published:** 2020-11-23

**Authors:** Nobuto Nakanishi, Jun Oto, Rie Tsutsumi, Yusuke Akimoto, Yuki Nakano, Masaji Nishimura

**Affiliations:** 1 Emergency and Critical Care Medicine, Tokushima University Hospital, 2-50-1 Kuramoto, Tokushima, 770-8503 Japan; 2 Department of Nutrition and Metabolism, Tokushima University Graduate School of Biomedical Sciences, 3-18-15 Kuramoto, Tokushima, 770-8503 Japan; 3 Intensive Care Medicine, Tokushima Prefectural Central Hospital, 1-10-3 Kuramoto, Tokushima, 770-8539 Japan

**Keywords:** Muscle atrophy, Upper limb, In-hospital mortality, Critically ill patients, Ultrasonography

## Abstract

**Background:**

Lower limb muscle atrophy is often observed in critically ill patients. Although upper limb muscles can undergo atrophy, it remains unclear how this atrophy is associated with clinical outcomes. We hypothesized that this atrophy is associated with mortality and impairments in physical function.

**Methods:**

In this two-center prospective observational study, we included adult patients who were expected to require mechanical ventilation for > 48 h and remain in the intensive care unit (ICU) for > 5 days. We used ultrasound to evaluate the cross-sectional area of the biceps brachii on days 1, 3, 5, and 7 and upon ICU discharge along with assessment of physical functions. The primary outcome was the relationship between muscle atrophy ratio and in-hospital mortality on each measurement day, which was assessed using multivariate analysis. The secondary outcomes were the relationships between upper limb muscle atrophy and Medical Research Council (MRC) score, handgrip strength, ICU Mobility Scale (IMS) score, and Functional Status Score for the ICU (FSS-ICU).

**Results:**

Sixty-four patients (43 males; aged 70 ± 13 years) were enrolled. The Acute Physiology and Chronic Health Evaluation (APACHE) II score was 27 (22–30), and in-hospital mortality occurred in 21 (33%) patients. The decreased cross-sectional area of the biceps brachii was not associated with in-hospital mortality on day 3 (*p* = 0.43) but was associated on days 5 (*p* = 0.01) and 7 (*p* < 0.01), which was confirmed after adjusting for sex, age, and APACHE II score. In 27 patients in whom physical functions were assessed, the decrease of the cross-sectional area of the biceps brachii was associated with MRC score (*r* = 0.47, *p* = 0.01), handgrip strength (*r* = 0.50, *p* = 0.01), and FSS-ICU (*r* = 0.56, *p* < 0.01), but not with IMS score (*r* = 0.35, *p* = 0.07) upon ICU discharge.

**Conclusions:**

Upper limb muscle atrophy was associated with in-hospital mortality and physical function impairments; thus, it is prudent to monitor it. (321 words)

**Trial registration:**

UMIN 000031316. Retrospectively registered on 15 February 2018.

**Supplementary Information:**

The online version contains supplementary material available at 10.1186/s40560-020-00507-7.

## Background

Muscle atrophy and weakness are serious problems in critically ill patients and can lead to post-intensive care syndrome [1]. As such, lower limb muscle atrophy is often observed in critically ill patients [2, 3]. In a previous study, after admission to the intensive care unit (ICU), the rectus femoris muscle atrophy was rapid, reaching 17.7% on day 10 [2]. Several studies have evaluated lower limb muscle atrophy [4, 5], but only few have focused on atrophy of upper limb muscles. The occurrence of upper limb muscle atrophy was considered disputable; a study reported that the upper limb muscle mass remained unchanged throughout the ICU course [6]. However, in our previous research, the upper limb muscle mass decreased by 13.2–16.9% over 7 days of ICU stay [7].

Although our previous study determined that upper limb muscles can undergo atrophy, it remains unclear how this atrophy is associated with mortality and physical functions. Upper limbs play an important role in critically ill patients, with their dysfunction frequently observed in ICU survivors [8]. Upper limbs can help mechanically ventilated patients perform activities and communication while on the bed [9]. Furthermore, upper limbs contribute to mobilization when the need to transfer or ambulate arises [10]. Despite these functions, limited data are available regarding the clinical outcomes of upper limb atrophy.

Because lower limb muscle atrophy has been associated with physical function impairment and mortality in previous studies [11–13], we hypothesized that upper limb muscle atrophy is also associated with mortality and functional impairments. Thus, in this study, we primarily investigated whether upper limb muscle atrophy was associated with in-hospital mortality. Second, we investigated the association between upper limb muscle atrophy and physical functions upon ICU discharge.

## Methods

This was a two-center prospective observational study at the mixed medical/surgical ICU of Tokushima University Hospital and Tokushima Prefectural Central Hospital from May 2016 to April 2019 (Table S1). We assessed upper limb muscle atrophy and physical functions on days 1, 3, 5, and 7 and upon ICU discharge. We additionally assessed lower limb muscle atrophy to perform comparisons. This study was approved by the clinical research ethics committee at Tokushima University Hospital and Tokushima Prefectural Central Hospital (approval numbers 2593 and 1739, respectively) and was registered as a clinical trial (University Hospital Medical Information Network Clinical Trials Registry: 000031316). Written informed consent was obtained from patients or surrogates at enrolment. A part of this research was previously reported in the form of a research letter [7].

### Study population

We included patients who were expected to require mechanical ventilation for > 48 h and to remain in the ICU for > 5 days and prospectively recruited them within 24 h of ICU admission on weekdays. We excluded patients who fell under one or more of the following categories: < 18 years of age, exhibiting trauma to or amputation of the upper or lower limbs, diagnosed with primary neuromuscular disease, and unclear ultrasound image.

### Nutrition and physiotherapy

Enteral nutrition was initiated within 24–48 h and was gradually increased according to the patients’ condition. Intravenous hyperalimentation was used only in patients who were not expected to receive enteral nutrition for prolonged periods. There were no protocolized nutritional goals of calorie and protein intakes. Physiotherapy was initiated within 48 h based on a progressive mobilization protocol described by Morris et al. [14] and was conducted by a multidisciplinary team including bedside nurses and physical therapists. During the study period, we recorded the calorie and protein intakes via the enteral and parenteral route as well as ICU Mobility Scale (IMS) scores.

### Ultrasound measurement

We used B-mode ultrasound, which was connected to a linear transducer. All scanning protocols were performed with the elbows and knees extended in the spine position. The dominant limb was then evaluated. Generous amounts of contact gel were applied to avoid muscle compression by the transducer. As described elsewhere [7, 15], the transducer was placed perpendicular to the long axis of the limbs. We evaluated the cross-sectional area, which was measured by outlining the muscle area shown in the transverse plane (Fig. S1), of the biceps brachii at two thirds the distance between the acromion and the antecubital crease (Fig. S2) and of the rectus femoris at midway between the anterior superior iliac spine and the proximal end of the patella. For consistency, each muscle measurement location was marked, and this mark was used for performing subsequent measurements. An ICU physician (N.N.) conducted the measurements three times, with the median value used for the evaluation after the confirmation of reliable correlation coefficients by two ICU physicians, intraclass value of 0.96–0.99, and interclass value of 0.99, as reported previously (Table S2) [7].

### Physical assessment

All patients, including those who had independent physical function before ICU admission and no traumatic or paralytic physical impairments upon ICU discharge, underwent physical assessment following at least three of De Jonghe’s five-command criteria [16]. Physical assessments were conducted by physical therapists who were blinded to ultrasound results. Limb muscle strength was evaluated using the Medical Research Council (MRC) score, which uses the sum of manual muscle strength at bilateral shoulder abductors, elbow flexors, wrist extensors, hip flexors, knee extensors, and ankle dorsiflexors [17]. ICU-acquired weakness (ICU-AW) was defined as an MRC score of < 48 on two separate occasions, and patients with a preadmission MRC score of < 48 were excluded [18]. Handgrip strength was measured using handgrip dynamometry (TKK 5401, Takei Scientific Instruments Co., Tokyo, Japan). Subjects performed handgrip tests twice using the dominant hand, and the higher value was used for evaluation. For handgrip tests, subjects either sat or lay in bed at 30°, with their elbows at 90° as previously reported [19]. IMS score and Functional Status Score for the ICU (FSS-ICU) were used to evaluate physical functions. IMS is a scale of mobilization capabilities ranging from 0 (lying in bed) to 10 (walking independently) [20]. FSS-ICU, on the other hand, consists of five functional tasks, including rolling, transfer from spine to sit, sitting at the edge of the bed, transfer from sitting to standing position, and walking [21]. Scores range from 0 (unable to attempt or complete task due to weakness) to 7 (complete independence).

### Outcomes

As the primary outcome, the ratio of upper limb muscle atrophy between survivors and non-survivors (death during the hospital stay) was determined and compared on days 3, 5, and 7. In the secondary analysis, we evaluated the relationship between upper limb muscle atrophy and MRC score, occurrence of ICU-AW, handgrip test results, IMS score, and FSS-ICU. For comparison, we conducted the same analysis for lower limb muscle atrophy.

### Sample size and statistical analyses

We enrolled a feasible sample size of approximately 60 based on a previous study [2, 13]. Continuous data were expressed as mean ± standard deviation or median (interquartile ranges [IQR]) and categorical data as number (%). Continuous variables were analyzed using the *t* test or Mann–Whitney *U* test, whereas categorical variables were analyzed using the chi-square test or Fisher’s exact test. A mixed-effect model for repeated measures was used to assess the changes in muscle mass over time, with time and subject as a fixed factor and a random factor, respectively. Logistic regression analyses were performed using age, sex, and Acute Physiology and Chronic Health Evaluation (APACHE) II score to evaluate the independent associations of in-hospital mortality. The APACHE II score was included in the analysis because the severity of illness affected upper limb muscle atrophy [7]. The models’ goodness of fit and discrimination ability were assessed using the Hosmer–Lemeshow test and the c-statistic, respectively. We constructed receiver operating characteristic (ROC) curves to determine whether the atrophy ratio could predict in-hospital mortality. Pearson’s correlation coefficient was used to assess correlations between muscle atrophy and functional impairments. We also analyzed the correlation between biceps brachii and rectus femoris muscle atrophy. Sensitivity analysis was conducted in sepsis defined by sepsis-3 criteria. Data analyses were conducted using JMP version 13.1.0 (SAS Institute Inc., Cary, NC, USA) and SPSS version 26.0 (IBM Corp., Armonk, NY, USA). All statistical tests were two-tailed, with the chosen type 1 error rate being *p* < 0.05.

## Results

**Fig. 1:**
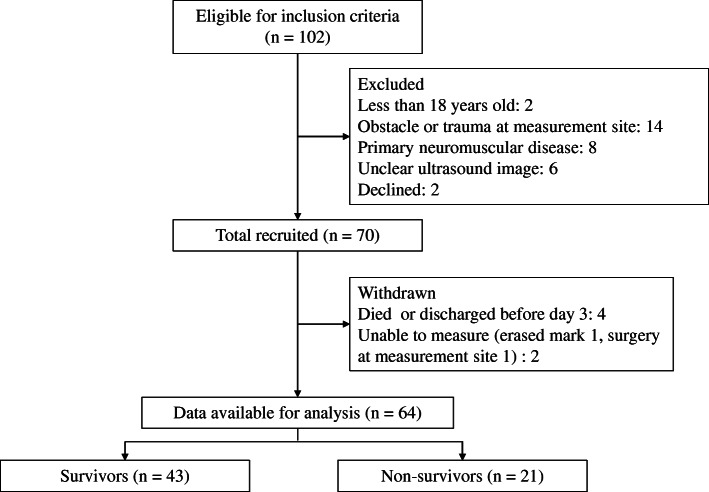
Flowchart of patients enrolled in this study. A total of 70 patients were recruited, and 64 were included in the analysis.

A total of 70 patients were enrolled (Fig. 1), and 64 patients remained in the study on day 3, 56 on day 5, and 36 on day 7. The age of the patients was 70 ± 13 years, and 43 (67%) were male (Table 1). The median APACHE II score was 27 (IQR, 22–30). Sepsis was present in 34 (53%) patients. The duration of mechanical ventilation was 6 days (IQR, 4–13 days), and ICU stay was 10 days (IQR, 6–16 days). The reasons for admission were respiratory failure (42%), nonrespiratory sepsis (14%), and postcardiac surgery (11%). During the study period, 34% and 14% patients received corticosteroids and continuous neuromuscular blocking agents, respectively. The calorie and protein intakes were 8.6–13.3 kcal/kg and 0.4–0.7 g/kg, respectively, and the median IMS score was 0–1 through 7 days of ICU stay.

**Table 1:** Patient characteristics

Variables	Overall (*n* = 64)
Age, years (mean [SD])	70 ± 13
Sex (men), *n* (%)	43 (67)
Body mass index, kg/m^2^	22.6 (20.4–25.9)
Acute Physiology and Chronic Health Evaluation II	27 (22–30)
Sequential Organ Failure Assessment	8 (5–10)
Sepsis defined by sepsis-3 criteria	34 (53)
Length of mechanical ventilation, days	6 (4–13)
Length of ICU stay, days	10 (6–16)
Length of hospital stay, days	38 (18–57)
Mortality in the ICU, *n* (%)	13 (20)
Mortality in the hospital, *n* (%)	21 (33)
ICU admission reasons, *n* (%)	
Respiratory failure	27 (42)
Sepsis, non-respiratory	9 (14)
Post-cardiac surgery	7 (11)
Heart failure	5 (8)
Trauma	5 (8)
Cardiac arrest	5 (8)
Neurologic	3 (5)
Other	3 (5)
Comorbidities^a^, *n* (%)	
Diabetes mellitus	15 (23)
Cancer	7 (11)
Chronic obstructive pulmonary diseases	2 (3)
Medications, *n* (%)	
Opioid	55 (86)
Midazolam	32 (50)
Dexmedetomidine	33 (52)
Propofol	18 (28)
Catecholamine^b^	40 (63)
Steroids^c^	22 (34)
Neuromuscular blocking agents^d^	9 (14)
Aminoglycoside	2 (3)
Nutrition^e^	
Calorie, kcal/kg	
Day 3	8.6 (3.5–13.1)
Day 5	13.1 (8.3–18.7)
Day 7	13.3 (8.0–17.3)
Protein, g/kg	
Day 3	0.4 (0.2–0.8)
Day 5	0.7 (0.3–1.0)
Day 7	0.6 (0.3–1.0)
ICU mobility scale	
Day 3	0 (0–1)
Day 5	0 (0–1)
Day 7	1 (0–1)

Data were presented as median (interquartile range) unless otherwise indicated

*SD* standard deviation, *ICU* intensive care unit

^a^Presence of comorbidities at the ICU admission

^b^Catecholamines including dopamine, dobutamine, noradrenaline, and adrenaline ^c^Corticosteroids with intravenous or peroral use

^d^Neuromuscular blocking agents with continuous use

^e^Enteral and parenteral nutrition

### Upper limb muscle atrophy

**Fig. 2:**
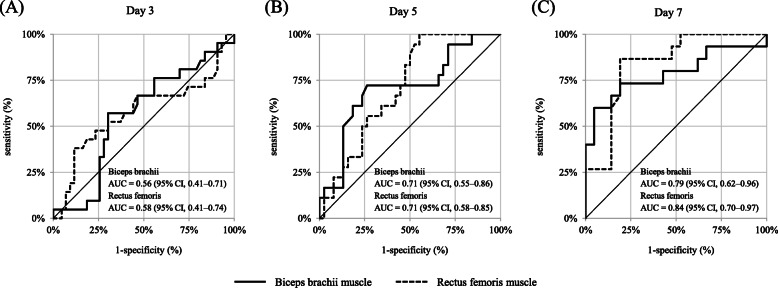
ROC curves of biceps brachii and rectus femoris muscle atrophy for the prediction of in-hospital mortality. The number of patients were 64, 56, and 36 on days 3, 5, and 7. Biceps brachii muscle atrophy showed no discriminative power of in-hospital mortality on day 3 (*p* = 0.70) but showed discriminative power on days 5 (*p* = 0.02; sensitivity, 0.72; specificity, 0.74 at a cut-off value of − 12.0%) and 7 (*p* = 0.01; sensitivity, 0.60; specificity 0.95 at a cut-off value of − 19.1%). Rectus femoris muscle atrophy showed no discriminative power of in-hospital mortality on day 3 (*p* = 0.53) but showed discriminative power on days 5 (*p* = 0.02; sensitivity, 1.00; specificity 0.45 at a cut-off value of − 9.5%) and 7 (*p* = 0.01; sensitivity, 0.87; specificity, 0.81 at a cut-off value of − 18.3%). The cut-off value was determined by the Youden index using JMP Statistical Software version 13.1.0 (SAS Institute Inc., Cary, NC, USA)

The cross-sectional area of the biceps brachii had decreased by 6.0% (95% CI, 4.4–7.6%) on day 3, 11.0% (95% CI, 9.3–12.7%) on day 5, and 15.6% (95% CI, 13.5–17.6%) on day 7 (*p* < 0.01). The ratio of survivors and non-survivors were 43/21, 38/18, and 21/15 on days 3, 5, and 7, respectively (patients’ characteristics are compared in Table S3). The muscle mass upon ICU admission had no significant effect on mortality (survivors vs. non-survivors, 5.9 vs. 5.1 cm^2^, *p* = 0.24 [univariate], *p* = 0.22 [multivariate]). On day 3, biceps brachii muscle atrophy was comparable between survivors and non-survivors (*p* = 0.43), but non-survivors had a higher degree of biceps brachii muscle atrophy on days 5 (*p* = 0.01) and 7 (*p* < 0.01, Table 2). In multivariate analysis including the severity of illness, a significant difference was noted on days 5 (*p* = 0.01) and 7 (*p* < 0.01). These associations on days 5 and 7 were also observed in sensitivity analysis including only sepsis (Table S4). As shown by ROC curves, the biceps brachii muscle atrophy did not predict in-hospital mortality on day 3 (*p* = 0.70, Fig. 2) but predicted mortality on days 5 and 7 (*p* = 0.02 and *p* = 0.01, respectively).

**Table 2:** Biceps brachii and rectus femoris muscle atrophy between survivors and non-survivors

	Day 3	Day 5	Day 7
	*n* = 43/21*	*n* = 38/18*	*n* = 21/15*
Biceps brachii muscle atrophy ratio			
Survivors (%)	5.8 (1.7–11.9)	10.8 (2.7–12.1)	12.2 (9.0–13.9)
Non-survivors (%)	8.0 (3.7–10.1)	14.8 (7.3–17.0)	19.6 (12.5–28.3)
Univariate analysis *p* value^a^	0.43	0.01	< 0.01
Multivariate analysis *p* value^b^	0.38	0.01	< 0.01
Rectus femoris muscle atrophy ratio			
Survivors (%)	6.1 (0.7–11.5)	11.0 (3.4–18.9)	15.6 (3.3–18.0)
Non-survivors (%)	11.1 (4.6–17.6)	18.0 (12.2–23.5)	23.9 (20.2–31.3)
Univariate analysis *p* value^a^	0.32	0.01	< 0.01
Multivariate analysis *p* value^b^	0.78	0.049	< 0.01

Atrophy ratio was reported as median (interquartile range)

*The number shows survivors/non-survivors in each study day

^a^Univariate analysis was conducted comparing the atrophy ratio between survivors and non-survivors

^b^Multivariate analysis was conducted using age, gender, APACHE II score, and the limb muscle atrophy to evaluate in-hospital mortality. Good fit was confirmed with the Hosmer–Lemeshow test (*p* = 0.28–0.85), and the c statistics was 0.71–0.93

Physical function was assessed in 27 patients upon ICU discharge. The median day of discharge was 6 days (IQR, 5–14 days). The median value of physical functions was as follows: MRC score, 51 (IQR, 44–59); ICU-AW, 33% (9/27); handgrip strength, 11.9 kg-force (IQR, 9.4–21.5); IMS score 2 (IQR, 1–4); and FSS-ICU, 9 (IQR, 2–17). Biceps brachii muscle atrophy was prominent in patients with ICU-AW compared with that in patients without ICU-AW (19.8% vs. 11.8%, *p* = 0.01). The MRC score, handgrip strength, and FSS-ICU were correlated with biceps brachii muscle atrophy, whereas the IMS score was not correlated with atrophy (Table 3).

**Table 3:** Correlation between biceps brachii and rectus femoris muscle atrophy and physical functions at ICU discharge

	ICU discharge (*n* = 27)	
	*r*	*p* value
Biceps brachii muscle		
Medical Research Council score	0.47	0.01
Handgrip strength	0.50	0.01
ICU Mobility Scale	0.35	0.07
Functional Status Score for the ICU	0.56	< 0.01
Rectus femoris muscle		
Medical Research Council score	0.46	0.02
Handgrip strength	0.62	< 0.01
ICU Mobility Scale	0.32	0.11
Functional Status Score for the ICU	0.56	< 0.01

Pearson correlation coefficient was used to assess the correlations

### Lower limb muscle atrophy

The rectus femoris cross-sectional area decreased by 6.2% (95% CI, 3.3%–9.1%) on day 3, by 12.9% (95% CI, 9.8–15.9%) on day 5, and by 17.1% (95% CI, 13.4%–20.7%) on day 7 (*p* < 0.01). Rectus femoris muscle atrophy was correlated with biceps brachii muscle atrophy on day 5 (*r* = 0.40, *p* < 0.01) and day 7 (*r* = 0.43, *p* = 0.01) but not on day 3 (*r* = 0.12, *p* = 0.36). The muscle mass upon ICU admission was not correlated to mortality (survivors vs. non-survivors, 5.2 vs. 4.4 cm^2^, *p* = 0.09 [univariate], *p* = 0.10 [multivariate]). Although rectus femoris muscle atrophy was comparable between survivors and non-survivors on day 3 (*p* = 0.32), non-survivors exhibited progressive rectus femoris muscle atrophy on days 5 and 7. In multivariate analysis, rectus femoris muscle atrophy differed significantly between survivors and non-survivors on day 5 (*p* = 0.049) and 7 (*p* < 0.01). As shown by ROC curves, rectus femoris muscle atrophy on day 3 did not predict in-hospital mortality (*p* = 0.53), and atrophy on days 5 and 7 predicted mortality (*p* = 0.02 and *p* = 0.01, respectively). Rectus femoris muscle atrophy was prominent in patients with ICU-AW compared with that in patients without ICU-AW (27.1% vs. 14.1%, *p* < 0.01). The MRC score, handgrip strength, and FSS-ICU were correlated with rectus femoris muscle atrophy, whereas the IMS score showed no such correlation.

## Discussion

In this study, we observed progressive upper limb muscle atrophy and found that it was associated with in-hospital mortality and functional impairments. To the best of our knowledge, this is the first study to report the progressive decline of upper limb muscle cross-sectional area and its associated clinical outcomes.

Although the occurrence of upper limb muscle atrophy is disputable, we confirmed upper limb muscle atrophy as with our previous research letter [7]. In a previous report, Turton et al. found that upper limb muscle thickness remained unchanged during the first 10 days of ICU admission (3.20 ± 0.58 cm to 2.98 ± 0.83 cm, *p* = 0.62), but these patients were less critical (APACHE II score, 23.0 vs. 27.0) [6]; hence, they may use their upper limbs while undergoing bed rest. However, in critically ill patients, upper limb atrophy was observed and was associated with in-hospital mortality, which is apprehensible because Puthucheary et al. reported muscle atrophy to be associated with multiorgan failure [2]. Because upper and lower limb muscle atrophy were weakly correlated on days 5 and 7, both atrophies may occur simultaneously in patients requiring prolonged stay.

Despite early muscle atrophy occurring on day 3, the early muscle atrophy did not affect mortality. Because our study included patients who were expected to stay in the ICU for > 5 days, day 3 was too early to predict survival. Patients’ survival may change after 3 days of treatment in the ICU. On the other hand, Hadda et al. reported that the degree of atrophy on day 3 can predict in-hospital mortality [13]. There are two possible reasons for this difference. First, we measured the muscle cross-sectional area, whereas the previous study measured muscle thickness. The former measurement is more reliable than the latter [12, 22]. Therefore, our measurement protocol is less susceptible to measurement bias. Second, we included mechanically ventilated patients, whereas the previous research included septic patients. Inflammation is the main cause of muscle atrophy in the early course of critical illness [23]. Catabolism is more prominent in septic than in non-septic patients [24]. Therefore, sepsis may have caused prominent muscle atrophy related with in-hospital mortality on day 3. Although our sensitivity analysis in 34 septic patients did not show an association between muscle atrophy on day 3 and in-hospital mortality, we cannot exclude the possible association due to the sensitivity analysis results performed in small numbers. Similarly, muscle mass upon admission did not predict in-hospital mortality, contrary to the findings of previous studies [25, 26]. Because of variations in muscle atrophy progression among patients, we considered muscle atrophy to be a more important predictor of mortality than muscle mass upon ICU admission, indicating that the muscle mass measurement from days 1 to 5 or 7 may contribute to the assessment of patients’ survival. Because many patients were discharged till day 7, it may be clinically useful to perform follow-up measurement on day 5, as suggested by Palakshappa et al. [27].

In the secondary outcomes, upper limb muscle atrophy was correlated with physical impairments upon ICU discharge. These correlations were limited to moderate correlations probably owing to the limited sample size. The IMS score was not significantly correlated with muscle atrophy. IMS was scored from 0 to 10 and was less fragmented than FSS-ICU (0–35 with 5 functional items) [28]. Atrophy assessment will be clinically useful for functional impairment assessment because functional tests can be performed only in conscious patients despite its clinical utility [29–31]. In a previous report, ICU-AW can be assessed in less than half of critically ill patients [32], but ultrasonographic assessment is applicable in almost all patients [15, 33].

Our finding on lower limb muscle atrophy is consistent with the findings of previous studies (Table 2) [2, 11, 13]. Because we included more critically ill patients, the atrophy ratio was more prominent than that in previous studies. Our study reported a 17.1% reduction through 7 days of ICU stay at APACHE II 27, Puthucheary et al. a 12.5% reduction at APACHE II 23.5, and Hadda et al. a 10.6% reduction at APACHE II 17 [2, 13]. Rectus femoris muscle atrophy was associated with in-hospital mortality, as reported previously [13, 24]. Moreover, the atrophy was correlated with physical function impairments as previous studies reported a moderate association between decreased rectus femoris cross-sectional area and physical functions (*r* = 0.71, *p* = 0.02) or MRC score (*r* = 0.51, *p* = 0.03) [11, 22]. Therefore, our research on upper limb muscle atrophy is reliable.

During the study period, protein intake and physiotherapy were limited due to the severity of critical illness. Recently, Nakamura et al. reported a protein intake of 1.5 g/kg/day and that enhanced physiotherapy prevented muscle atrophy [34]. Sufficient nutrition and physiotherapy are essential for preventing muscle atrophy. Enhanced training of upper limbs may improve mortality and physical impairment related to muscle atrophy. In critically ill patients, lower limb training is predominantly the rehabilitation focus based on the concern for early mobilization [35, 36]. However, in recent studies, neuromuscular electrical stimulation has been additionally used for upper limb training [37–39], preventing upper limb muscle atrophy, and the enhanced upper limb training reduced the length of hospitalization [39]. Our findings suggest that it is worthwhile to monitor upper limb as well as lower limb muscle atrophy and perform early interventions to improve the quality of life in critically ill patients.

## Limitations

Our study has several limitations. First, the sample size was small. Therefore, multivariate analysis only included age, sex, and APACHE II score, although comorbidities, medications, and presence of sepsis may affect muscle atrophy. Second, it is not completely fair to compare the measurement days because the number of patients in the ICU differed from day 3 to day 7, although most patients remained in the ICU till day 5. Third, physical function was assessed only upon ICU discharge because insufficient consciousness hampered the assessment of physical function during ICU stay. Fourth, we assessed the correlation of ultrasound measurement but did not assess the correlation of physical assessments because there were conducted as part of clinical practice.

## Conclusions

We evaluated upper limb muscle atrophy and found that it was associated with in-hospital mortality and physical function impairment; thus, it is prudent to monitor upper limb muscle atrophy.

## Supplementary Information

**Additional file 1:** **Figure S1.** Image of ultrasound. **Figure S2** Measurement sites of ultrasound. **Table S1** Facility and equipment in this two-center prospective observational study. **Table S2** Reproducibility of measurements. **Table S3** Patient characteristics between survivors and non-survivors. **Table S4** Biceps brachii and rectus femoris muscle atrophy between survivors and non-survivors in sepsis defined by sepsis-3 criteria.

## Data Availability

The datasets generated during the current study are available from the corresponding author on reasonable request.

## Abbreviations

ICU: Intensive care unit
MRC: Medical Research Council
ICU-AW: ICU-acquired weakness
IMS: ICU mobility scale
FSS-ICU: Functional Status Score for the ICU
IQR: Interquartile ranges
APACHE: Acute Physiology and Chronic Health Evaluation
ROC: Receiver operating characteristic
