# Supplementary material for: Upper limb muscle atrophy associated with in-hospital mortality and physical function impairments in mechanically ventilated critically ill adults: a two-center prospective observational study
Source: J Intensive Care. 2020 Nov 23;8:87. doi: 10.1186/s40560-020-00507-7 (PMC7684934; doi:10.1186/s40560-020-00507-7)
Supplement: Supplementary file 1 — Additional file 1: Figure S1. Image of ultrasound. Figure S2 Measurement sites of ultrasound. Table S1 Facility and equipment in this two-center prospective observational study. Table S2 Reproducibility of measurements. Table S3 Patient characteristics between survivors and non-survivors. Table S4 Biceps brachii and rectus femoris muscle atrophy between survivors and non-survivors in sepsis defined by sepsis-3 criteria. [file 40560_2020_507_MOESM1_ESM.docx]

Supplemental File

Upper limb muscle atrophy associated with in-hospital mortality and physical function impairments in mechanically ventilated critically ill adults: A two-center prospective observational study

**Table S1.** **Facilities and equipment in this two-center prospective observational study**

|  | **Tokushima University Hospital** | **Tokushima Prefectural Central Hospital** |
| --- | --- | --- |
| Hospital type | Tertiary hospital | Tertiary hospital |
| Number of hospital beds | 692 | 460 |
| Annual hospital admissions | 14,000 to 15,000 | 11,000 to 12,000 |
| ICU type | Closed | Open |
| ICU admission type | mixed medical/surgical ICU | mixed medical/surgical ICU |
| Number of ICU beds | 10 | 8 |
| Annual ICU admissions | 400 to 500 | 400 to 500 |
| Ultrasounds | HI VISION Preirus, Hitachi Medical Corporation, Tokyo, Japan | LOGIQ P9, GE healthcare, WI, USA |
| Transducers | EUP-L73S liner transducer, Hitachi Medical Corporation, Tokyo, Japan | 12L-RS liner transducer, GE healthcare, WI, USA |

**Figure S1** Image of ultrasound

**
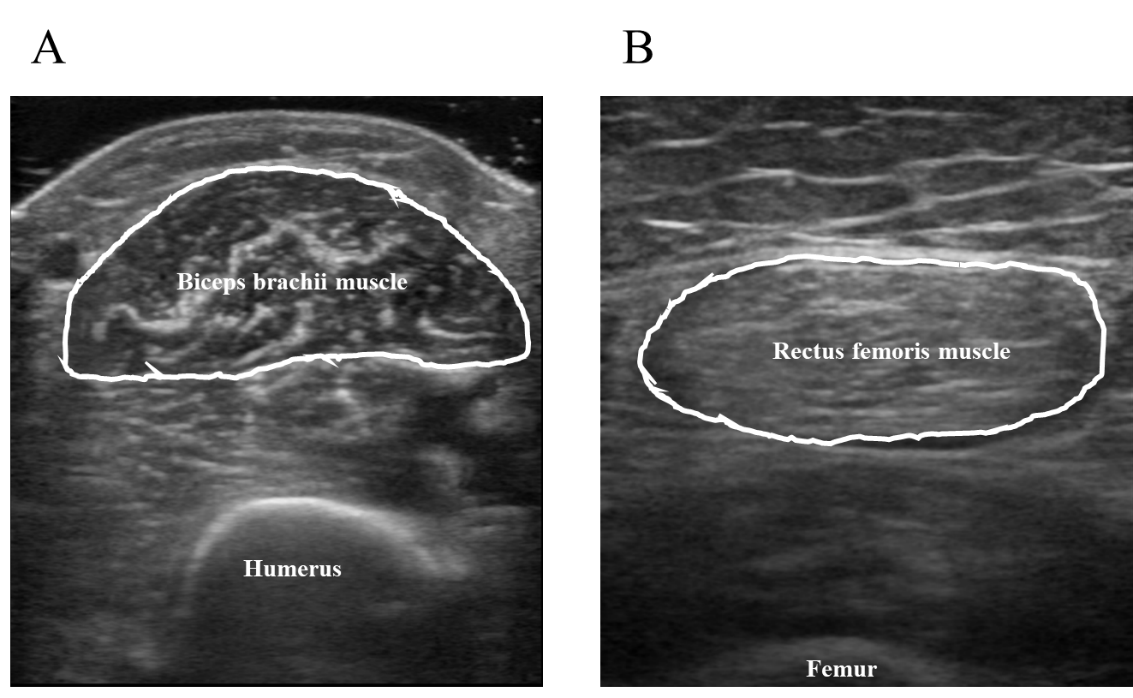
**

A. The cross-sectional area of biceps brachii was measured by tracking the muscle area shown in the transverse plane. B. The cross-sectional area of rectus femoris was measured by tracking the muscle area shown in the transverse plane.

**Figure S2** Measurement sites of ultrasound

**
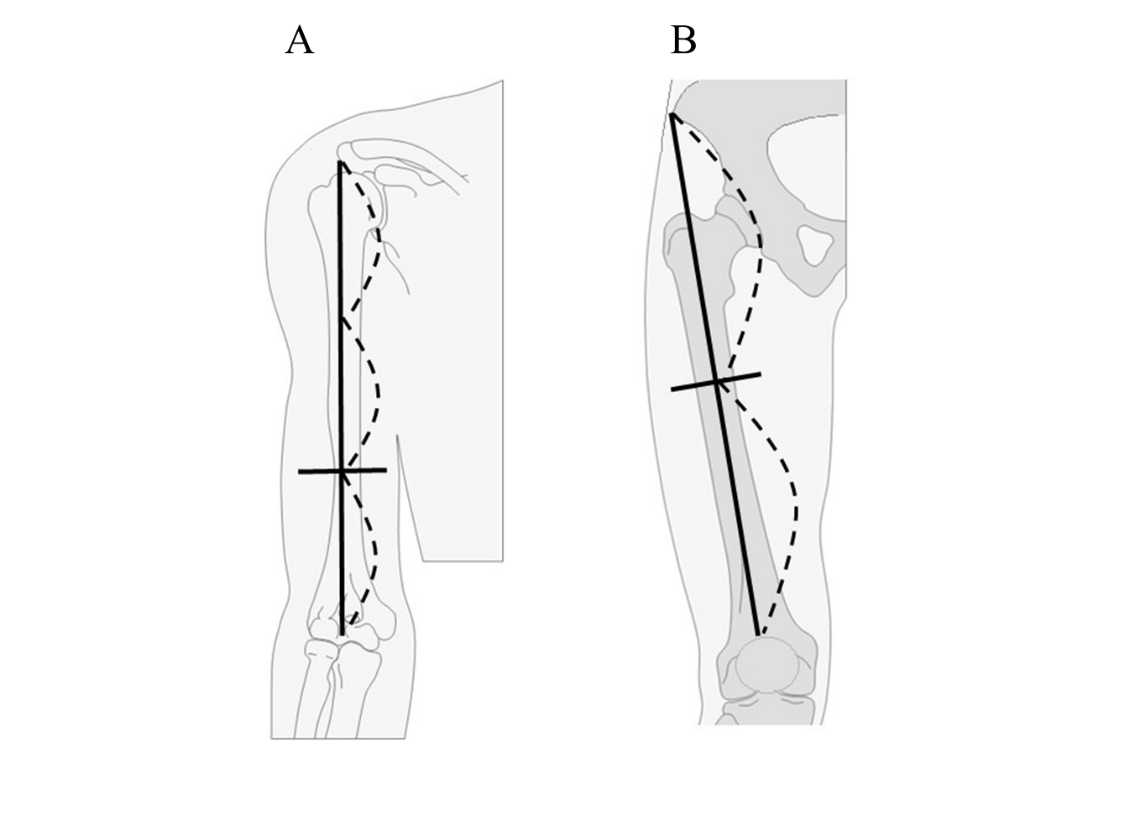
**

A. Biceps brachii muscle was measured at two-thirds of the way between the acromion and the antecubital crease. B. Rectus femoris muscle mass was measured at midway between the anterior superior iliac spine and the proximal end of the patella.

| **Table S2** Reproducibility of measurements | |  |  |  |  | |
| --- | --- | --- | --- | --- | --- | --- |
|  | Correlation coefficient | |  | Bland-Altman 95% CI | | |
| Variables | r | *p* |  | Bias | | 95% CI |
| Intra-observer reproducibility |  |  |  |  | |  |
| Biceps brachii cross-sectional area | 0.96 | < 0.01 |  | 0.028 ± 0.094 | | −0.184 to 0.240 |
| Rectus femoris cross-sectional area | 0.99 | < 0.01 |  | 0.113 ± 0.067 | | −0.039 to 0.265 |
| Inter-observer reproducibility |  |  |  |  | |  |
| Biceps brachii cross-sectional area | 0.99 | < 0.01 |  | 0.004 ± 0.075 | | −0.165 to 0.173 |
| Rectus femoris cross-sectional area | 0.99 | < 0.01 |  | 0.059 ± 0.076 | | −0.113 to 0.231 |

CI = confidence interval

Reproducibility was assessed for 10 patients before the study. The Pearson correlation coefficient and Bland-Altman plot were determined by using JMP statistical software version 13.1.0 (SAS Institute Inc., Cary, NC, USA).

**Table S3** Patient characteristics between survivors and non-survivors

| Variables | survivors | non-survivors | *p* value |
| --- | --- | --- | --- |
| Day 3 (n = 43, 21) |  |  |  |
| Age, years (mean [SD]) | 69 ± 12 | 72 ± 13 | 0.31 |
| Sex (Men), n (%) | 29 (67%) | 14 (67%) | 0.95 |
| Body mass index, kg/m^2^ | 22.7 (20.8–25.7) | 22.2 (19.4–26.3) | 0.48 |
| Sequential Organ Failure Assessment | 7 (5–10) | 10 (7–12) | 0.02 |
| Acute Physiology and Chronic Health Evaluation II | 25 (20–30) | 28 (26–38) | 0.01 |
| Length of ICU stay, days | 7 (5–11) | 15 (7–24) | 0.01 |
| Length of mechanical ventilation, days | 6 (4–11) | 13 (6–22) | 0.01 |
| Length of hospital stay, days | 42 (25–65) | 27 (13–46) | 0.07 |
| Day 5 (n = 38, 18) |  |  |  |
| Age, years (mean [SD]) | 68 ± 13 | 71 ± 14 | 0.47 |
| Sex (Men), n (%) | 26 (68%) | 12 (67%) | 0.90 |
| Body mass index, kg/m^2^ | 22.7 (20.8–25.8) | 23.3 (20.0–27.0) | 0.99 |
| Sequential Organ Failure Assessment | 7 (5–10) | 10 (8–13) | < 0.01 |
| Acute Physiology and Chronic Health Evaluation II | 25 (20–29) | 29 (26–41) | < 0.01 |
| Length of ICU stay, days | 9 (6–13) | 18 (11–27) | < 0.01 |
| Length of mechanical ventilation, days | 6 (4–12) | 14 (10–24) | < 0.01 |
| Length of hospital stay, days | 43 (25–59) | 32 (15–48) | 0.27 |
| Day 7 (n = 21, 15) |  |  |  |
| Age, years (mean [SD]) | 67 ± 13 | 72 ± 11 | 0.22 |
| Sex (Men), n (%) | 16 (76%) | 11 (73%) | 0.85 |
| Body mass index, kg/m^2^ | 22.6 (20.3–25.4) | 22.5 (20.0–27.7) | 0.62 |
| Sequential Organ Failure Assessment | 8 (4–10) | 10 (8–14) | 0.01 |
| Acute Physiology and Chronic Health Evaluation II | 27 (23–30) | 28 (25–42) | 0.14 |
| Length of ICU stay, days | 11 (10–17) | 21 (12–34) | 0.01 |
| Length of mechanical ventilation, days | 11 (6–16) | 15 (12–25) | 0.01 |
| Length of hospital stay, days | 50 (30–79) | 36 (15–53) | 0.12 |

| **Table S4** Biceps brachii and rectus femoris muscle atrophy between survivors and non-survivors in sepsis defined by sepsis-3 criteria | | | |
| --- | --- | --- | --- |
|  | Day 3 | Day 5 | Day 7 |
|  | n = 16/18* | n = 15/16* | n = 6/14* |
| Biceps brachii muscle atrophy ratio |  |  |  |
| Survivors (%) | 3.9 (1.7–7.4) | 10.4 (2.2–18.1) | 9.4 (5.0–14.9) |
| Non-survivors (%) | 7.1 (2.3–9.7) | 15.2 (9.1–17.1) | 19.4 (12.2–28.7) |
| Univariate analysis *p* value† | 0.19 | 0.10 | 0.02 |
| Multivariate analysis *p* value‡ | 0.21 | 0.04 | 0.02 |
| Rectus femoris muscle atrophy ratio |  |  |  |
| Survivors (%) | 5.8 (3.6–12.8) | 16.8 (−3.8–23.1) | 14.3 (1.6–18.8) |
| Non-survivors (%) | 9.9 (4.4–17.6) | 18.5 (12.1–23.8) | 24.0 (21.0–32.2) |
| Univariate analysis *p* value† | 0.44 | 0.15 | < 0.01 |
| Multivariate analysis *p* value‡ | 0.66 | 0.20 | < 0.01 |
| Atrophy ratio was reported as median (interquartile range).  *The number shows survivors/non-survivors in each study day. †Univariate analysis was conducted comparing the atrophy ratio between survivors and non-survivors. ‡Multivariate analysis was conducted using age, gender, APACHE II score, and the limb muscle atrophy to evaluate in-hospital mortality. Good fit was confirmed with the Hosmer-Lemeshow test (*p* = 0.31–0.86), and the c statistics was 0.68–0.95. | | | |
